# Right Testicular Artery Occlusion and Acute Appendicitis by *Angiostrongylus costaricensis*

**DOI:** 10.1155/2019/5670802

**Published:** 2019-08-27

**Authors:** Luis Enrique Sánchez-Sierra, Roberto Antonio Martínez-Quiroz, Héctor S. Antúnez, Humberto Cabrera-Interiano, Fernando Josué Barrientos-Melara

**Affiliations:** ^1^Instituto Hondureño de Seguridad Social, Honduras; ^2^Pediatric Surgery Service, Hospital Escuela Universitario, Honduras; ^3^Hospital Escuela Universitario, Honduras; ^4^Universidad Nacional Autónoma de Honduras, Honduras

## Abstract

*Introduction. Angiostrongylus costaricensis* is a nematode from the superfamily Metastrongyloidea, whose etymology is “roundworm that lives in blood vessels”. This parasite can be found from the southern United States to northern Argentina and southern Brazil. In 1983, Morera and Ruiz published the first case of a testicular artery occlusion by *A. costaricensis*. *Case Presentation*. A five year old boy presented with eight days of pain, denying trauma backgrounds and followed with an increase of volume. The treatment was a right simply orchiectomy, finding necrosis of the testicle, the biopsy showed reddish-purple aspect and soft consistency. Histologic studies reveled the presence of a worm inside the testicular artery. *Conclusion*. The diagnosis of *A. costaricensis* infection should be considered in all pediatric patients, with signs and symptoms of orchitis or acute abdomen, from endemic areas, may cause occlusion of the testicular artery and appendicular artery causing testicular and cecal appendix necrosis, respectively, even putting the patient's life at risk. The diagnosis is complex, because the clinical manifestations are similar to an orchitis or acute abdomen, therefore, the definitive diagnosis is made during the surgical intervention and histopathological study.

## 1. Introduction

Angiostrongyliasis is a parasite infection produced by 2 species of the genus *Angiostrongylus: Angiostrongylus costaricensis* [1], and *Angiostrongylus cantonensis,* that causes abdominal angiostrongyliasis and eosinophilic meningoencephalitis, respectively [2]. The *Angiostrongylus costaricensis* is a nematode from the superfamily Metastrongyloidea [3], whose etymology is “roundworm that lives in blood vessels” [4]. This parasite can be found from the southern United States to northern Argentina and southern Brazil [5], as well as Cuba, Puerto Rico and the Dominican Republic [4].

In 1905, Kamensky described the genus *Angiostrongylus* [6]. The first clinical characteristics of abdominal angiostrongyliasis were reported in 1952 in a boy from Costa Rica [7, 8], but the clinical syndrome was described on 1967 by Morera and Céspedes, which in 1971 was identified as a new species the *A. costaricensis* [7].

In 1972, Sierra and Morera, published the first case in Honduras and outside of Costa Rica [9]. In 1983 Morera and Ruiz, published the first case of a testicular artery occlusion by *A. costaricensis* in an 8 year old boy [10].

The objective of this case report is to describe the clinical evolution, diagnostic and treatment of a pediatric patient with an occlusion of the right testicular artery and acute appendicitis induced by *A. costaricensis*, which is a very rare presentation of the disease.

## 2. Case Presentation

A 5-year old boy is presented by his parents to Pediatric Surgery Emergency of the Hospital Escuela Universitario (HEU) in Tegucigalpa, Honduras; with a 8-day history of pain in his right testicle, with sudden onset, continuous, intense enough to prevent him from wandering around, denying trauma backgrounds, followed by an increase of the volume with same amount of time; vomiting preceded by nausea, 3-day vomiting evolution with 4 daily episodes of alimentary content; concomitantly, 4-day history of high fever, without a timetable predominance, attenuated with acetaminophen.

Physical examination of genitals showed an augmentation in size of the right testicle in comparison with the left one, inflammatory changes, tenderness, positive Prehn sign, bilateral cremasteric reflex present.

Blood count showed leukocytosis with eosinophilia (Table 1). Sonogram reported a testicle of 1.8×1.0×1.1 cm, volume of 1.07 mL, with alteration on its axis and hypoecogenic areas with irregular appearance, no sign of abstraction of discharge to color Doppler, nor power Doppler; showed diffuse enlargement of the epididymis, edema and inflammatory changes on the scrotal bag; no hydrocele nor pyocele. Left testicle with normal size and form.

The treatment was a right simple orchiectomy, finding necrosis of the testicle and pyocele. The biopsy showed a reddish-purple aspect and soft consistency testicle, followed by the spermatic cord, with macroscopic outbreaks of necrosis and softening zones.

**Figure 1:**
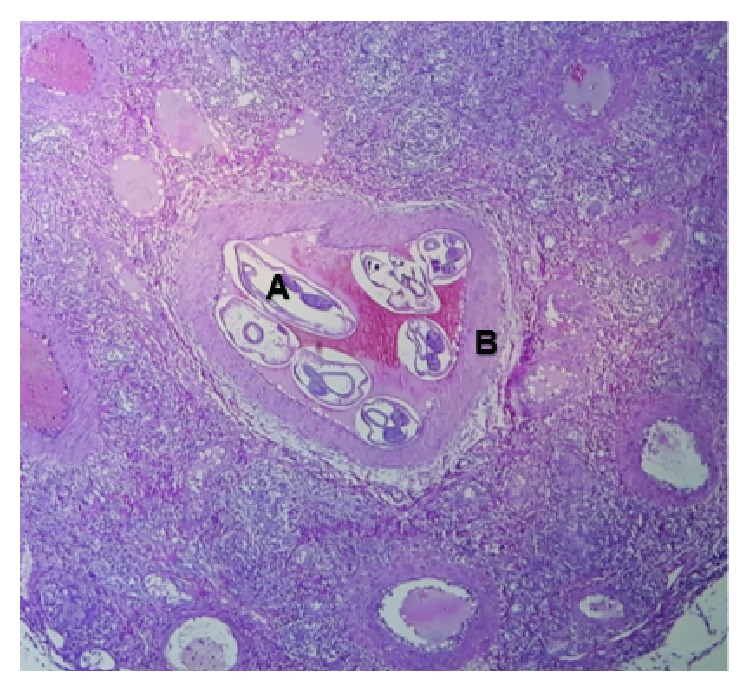
(A). *Angiostrongylus costaricensis* worm in the testicular artery lumen. (B). Blood vessel wall.

Histologic studies revealed the presence of a worm inside the testicular artery (Figure 1), surrounded by granulomatous inflammation with abundant eosinophils, testicular parenchyma showed wide discharges of hemorrhagic necrosis, eggs were not identified. Medical discharge was given 24 hours later with successful progress.

Patient presented to the Pediatric Surgical Emergency department 10 days after his last admission with a 7-day history of not having bowel movements, treated with 2 enemas without stimulating defecation; also 5-day evolution abdominal pain, located in the epigastrium, irradiated to the right iliac fossa, intense, attenuated with ibuprofen, 4-day history of fever, subjectively high, predominant at night. 3-day vomiting history with 4 daily episodes of dietary content.

During physical examination, abdomen was bloated with no umbilical scar deviation, to auscultation bowel sounds were absent, to percussion timpanums was confirmed, abdominal girth was 53 cm with tenderness and pain were located at the lower right quadrant, Blumberg and Rovsing signs were positive, no visceromegaly nor abdominal masses were found. Complete blood count presented increased WBC with eosinophilia (Table 1).

A laparoscopic appendicectomy was performed finding inflammatory changes in cecal appendix with small pinpoint marks in terminal ileum, caecum and appendix. The cecal appendix biopsy reported secondary eosinophilical appendicitis infection by *A. costaricensis,* secondary vascular occlusion and coagulative necrosis subsequently. Cecal appendix showed similar changes to the testicle, with worm in the arteriolar lumen, *A. costaricensis* eggs were found in capillaries, stroma and cecal appendix wall.

## 3. Discussion

In 1980 in Costa Rica, three important points were shown in relation to this disease: increase on the number of cases between August and November [11–13], the heaviest months of rain; the humid climate facilitates the appearance of the intermediate host, the slug, and it increases the risk of getting infected [11]*;* the pediatric population shows the highest infection rates, which could be explained by children's habit to put things in their mouth [14], the most affected are the grade-schoolers, especially boys because they play frequently outside their home and expose to slugs [13]. Longitudinal study in Honduras during 3 years determined epidemiological factors such as: warm weather, rain and broadleaf vegetation, major incidence between August and December and male gender [2, 4]. This case coincides with the cited literature, inasmuch as it was presented in August, in a 5-year-and-8-month old boy. The route of infection could be by contaminated food intake or direct ingestion of slugs.

In Honduras, cases of abdominal angiostrongyliasis have been reported coming from the departments of Cortés, Comayagua, Olancho, Francisco Morazán and El Paraíso [15]. The first case described outside Costa Rica was in Honduras in 1972 [9]. The first case of testicular artery occlusion by *A. costaricensis* was in 1983 in Costa Rica [10]. This patient came from Comayagua and it was considered, to be the first case published in Honduras about testicular artery occlusion secondary to *A. costaricensis* and the first to both clinical pictures regional level.

The human is an incidental host because he accidentally eats slugs, fruits or vegetables contaminated with mucoid secretions of mollusks [16–18]. Foods are infected with larvae L3, which behave in humans the same way as in rodent intestines [19], but the parasite does not complete its life cycle in humans [6]. This patient presented several typical characteristics of this disease such as: age with high prevalence of infection, tendency to play in the garden and putting objects repeatedly in his mouth.

The accumulation of eggs in the arteries and mesenteric arterioles with an intense eosinophilic response reduces the blood perfusion causing ischemic tissue necrosis [6], manifesting as acute abdomen, chronic abdominal pain and fever with eosinophilia [16]. In this patient the histopathological study showed worms in the testicular artery and in the ileocolic region, with an intense eosinophilical reaction, which caused occlusion to the arterial lumen, leading to ischemia and tissue necrosis to the testicular parenchyma and appendicular wall, respectively.

The most frequent location of the lesions due to *A. costaricensis* is around the cecal appendix, ileum, caecum and ascending colon, the extraintestinal tissues or ectopic locations that can be found with lesions are: lymph nodes, omentum, liver and less frequently the testes [7, 11, 13]. In this case, two target organs were affected, one frequent as the cecal appendix and one infrequent as the testicle, but both have in common their origin of arterial irrigation.

Macroscopically the intestinal wall is thickened, with whitish or yellowish granulomas, small white pinpoints in subserosa, with edema, causing partial or total obstruction of the intestinal lumen, it can progress to bleeding, necrosis and perforation [7, 13, 16]. In a microscopic plane the lesions appear as thickening of the intestinal wall, granulomatous inflammation with eosinophilic infiltration, surrounding the eggs of *A. costaricensis* [13, 18]. Histological sections of the mesentery show the presence of the worms in the lumen of the mesenteric artery and arterioles, it can cause arteritis and thrombosis lesions, with hypoperfusion and ischemic tissue necrosis [6]. There is two non-exclusive patterns of macroscopic lesions, both showing segmental distribution, were observed: a hypertrophic-pseudoneoplasic pattern (HP) involving predominant thickening of the intestinal wall and an ischemic-congestive pattern (IC) involving necrotic and/or congested areas [20]. This case, testicular parenchyma necrosis was observed macroscopically, the biopsy showed the violet reddish testicle and soft consistency. The histological study showed a worm inside the testicular artery, surrounded by granulomatous inflammation with abundant eosinophils, testicular parenchyma with foci of hemorrhagic necrosis, without eggs. Macroscopically, the appendix presented inflammatory changes with small punctate patches distributed in the terminal ileum and caecum. The biopsy reported eosinophilic appendicitis secondary to infection with *A. costaricensis*, secondary vascular occlusion and necrosis.

Obstruction of the testicular artery manifests with pain at 24 hours of evolution, in addition to blushing, heat and increased testicular volume [10]. This case presented a clinical picture of 8-days evolution right testicle pain, intense, limited wandering, with increased volume and flushing; positive Prehn's sign, plus a 3-day evolution fever.

The clinical manifestations of abdominal angiostrongyliasis are depressible abdomen with voluntary muscular resistance, spontaneous abdominal pain, hypersensitivity in iliac fossa and right flank, sign of positive rebound, accompanied by a mass in the lower right quadrant, fever, anorexia, vomiting [16, 18, 19] and painful digital rectal examination in 50% of cases [11]. This patient presented 7-days history of not having bowel movements, 5-days colic abdominal pain evolution, located in epigastrium with irradiation to right iliac fossa, intense; 4-day fever and 3-day vomiting evolution with 4 daily episodes of alimentary content. He presented a distended abdomen with a perimeter of 53 cm, absent bowel sound, tympanism, Blumberg and Rovsing sign positive, no abdominal masses were felt.

The blood count showed moderate leukocytosis and eosinophilia about 30-50% [3, 12], there are cases up to 60% [21]. The general examination of feces (EGH) was normal, imaging studies are not useful. Serology studies can be performed by ELISA; the Morera test is the most used, it is positive in more than 99% of cases [6]. Currently it is not available in Honduras [15]. Aramburú da Silva et al. In 2003, published that a PCR-based molecular diagnostic test for abdominal angiostrongyliasis has a huge potential for clinical use and for epidemiological studies [22]. The blood count of this patient coincides with the cited literature, showed leukocytosis of 16X10/uL, with eosinophilia of 44% suggesting parasitic infection, EGH showed no alterations. Serology by ELISA and Morera test was not performed, but it was confirmed with the histopathological study, showing the presence of the parasite.

The ultrasound findings in these patients are frequently edema and distention of intestinal loops in a diffuse form, with intraluminal fluid, granuloma or pseudonimus image in the ileocecal area [11]. The definitive diagnosis is confirmed by the identification of the *A. costaricensis* associated with granulomatous perivascular reaction, infiltrate and eosinophilic vasculitis in the histopathological examination of the surgical specimens; since there is no elimination of eggs or vermes in human feces [7, 16].

The differential diagnosis must be made with testicular torsion [10, 12, 15], because *A. costaricensis* migrates through the abdominal aortic artery to the right or left testicular artery, causing occlusion, subsequently ischemia and necrosis of the testicular parenchyma [7]. Abdominal angiostrongyliasis can simulate a case of acute appendicitis, because the *A. costaricensis* migrates through the abdominal aortic artery, superior mesenteric artery, right colic artery and the appendicular branch [7]. The patient was admitted and operated with preoperative diagnosis of testicular torsion due to clinical manifestations and lesions of necrosis in the testicle, the definitive diagnosis was made by histopathology, later was admitted due to suspected acute appendicitis secondary to *A. costaricensis* infection, considering his history of infection.

Different drugs have been tested in humans, but none have been found beneficial; treatment of choice is surgical [4], and in cases of acute abdomen due to necrosis or perforation of a target organ [15].

## 4. Conclusion

The diagnosis of *A. costaricensis* infection should be considered in all pediatric patients, with signs and symptoms of orchitis or acute abdomen, from endemic areas, may cause occlusion of the testicular artery and appendicular artery causing testicular and cecal appendix necrosis, respectively, even putting the patient's life at risk.

The diagnosis of angiostrongyliasis is complex, because the clinical manifestations are similar to an orchitis or acute abdomen, therefore, the definitive diagnosis is made during the surgical intervention and histopathological study, observing the worms; surgical treatment is not necessary in cases where there is no necrosis or perforation of any organ.

## Figures and Tables

**Table 1:** Blood count no. 1 is correlated with testicular artery occlusion. Blood count no. 2 is correlated with appendicitis. Both disease patterns are secondary to infection by *A. costaricensis.*

Parameter	Unit	Blood count no.1	Blood count no.2
Hemoglobin	g/dL	12.9	11.8

Hematocrit	%	37.4	32.6

Platelets	×10/*μ*L	314	314

White blood cells	×10/*μ*L	16.5	14.11

Neutrophils	%	37.2	66.5
	×10/*μ*L	6.15	9.38

Lymphocytes	%	13.6	9.3
	×10/*μ*L	2.25	1.31

Monocytes	%	2.9	4.4
	×10/*μ*L	0.49	0.63

Eosinophils	%	44.3	17.1
	×10/*μ*L	7.33	2.42

Basophils	%	0.5	0.3
	×10/*μ*L	0.09	0.05
